# Variation in behavioral preference and calcium binding expression in two *Synodontis* catfishes with different communication modalities

**DOI:** 10.3389/fnana.2025.1589687

**Published:** 2025-08-14

**Authors:** Carlos Daniel Corrales Parada, Iva Udovičić, Giulia Haschei, Boris Philippe Chagnaud

**Affiliations:** Department of Biology, University of Graz, Graz, Austria

**Keywords:** *Synodontis*, calcium binding proteins, parvalbumin, calretinin, calbindin, social communication, neuroanatomy, social preference

## Abstract

Animals use different communication modalities for social interactions, often showing sensory adaptations linked to their preferred signaling system. How such adaptations affect individual processing centers usually remains elusive due to interspecies differences. One system in which such adaptations can be investigated are *Synodontis* catfish. *Synodontids* generally use acoustic signals for social communication, but in some species, they generate electric signals. This allows to investigate adaptations of networks associated with social signal detection in closely related species. We investigated potential sensory adaptations in two *Synodontis* species (*Synodontis grandiops* - SG and *Synodontis nigriventris* - SN) with different communication channels. We tested their behavioral preferences toward different sensory modalities and found strong preferences for conspecifics. To investigate potential adaptations at the cellular level, we focused on the torus semicircularis (TS), a major midbrain sensory hub for auditory and electric sensory processing. We found an increase in projections from the anterior tuberal nucleus (AT) to the lateral TS (TSl, which processes electrosensory information) in SN, but no difference in the projections from the central TS (TSc) to AT in either species. An enhanced density of calcium binding proteins in the TSl was found only in SN. As electrocommunication is a derived communication channel in *Synodontis*, our findings suggest that a shift to electric communication may have led to (i) stronger projections to and from sensory regions, and (ii) a change in neurochemical profile, which together might facilitate social signal detection.

## Introduction

Sensory systems allow animals to extract information about the environment, including signals from conspecifics. Coupled together with signal production this provides the basis of social communication (Marler, 1967; Bradbury and Vehrencamp, 2011; Hebets et al., 2016). Communication signals originate from a wide diversity of physical modalities including, but not limited to, visual, chemical, tactile, acoustic, and electrical (Bradbury and Vehrencamp, 2011; Ward et al., 2020). Depending on the relative importance of a given sensory modality for different species and individuals, sensory systems, including their respective neural substrates, are differentially developed. Examples of such neural adaptations are the enlargement of pallial regions to process visual displays in manakins (Day et al., 2011), the appearance of a derived subdivision in the extrolateral nucleus and its general enlargement in mormyrid fish to process electrocommunication signals (Carlson et al., 2011), the addition of pallial regions for acoustic mate preferences in songbirds (Van Ruijssevelt et al., 2018), or the formation of specific cortical regions to process somatosensory information in mice (Petersen, 2007). Despite changes in size or the addition of novel brain areas, adaptations arising from a change to a new behavioral phenotype, such as a different communication modality or a different social behavior, could also appear (i) in neuronal processing (Bass, 1992; Kéver et al., 2020, 2021), (ii) via changes in connectivity by increasing innervation of relevant brain areas (Bass, 1992; Sisneros and Bass, 2003; Rohmann and Bass, 2011; Forlano et al., 2015; Ghahramani et al., 2015; Bhandiwad et al., 2017) or (iii) via changes in neurochemistry. The latter can be achieved, for instance, by either increasing the presence of neurotransmitters [e.g., dopamine and galanin (Ghahramani et al., 2015; Tripp et al., 2020)], or by increasing the presence of receptors or ion channels (Forlano et al., 2005; Forlano and Bass, 2011; Ghahramani et al., 2015; Tripp et al., 2018; Merritt et al., 2020; Tripp et al., 2020; Loveland et al., 2022).

A major problem in understanding which changes in neural circuits are associated with changes in communication modality is interspecies comparability. The manifold differences between species and their ways to produce and process communication signals make it difficult to directly compare neural circuits, and to identify key adaptations in social communication. More closely related species likely display less variability, and thus offer experimental advantages. One family that shows changes in communication modality in closely related species are *Synodontis* catfishes. Like all catfishes, they have an acoustic and a passive electrosensory system (Knudsen, 1976b; Bullock and Heiligenberg, 1986). While all species apparently produce sounds with their pectoral stridulation mechanism (Kaatz et al., 2025), they also include species that communicate through another sound producing mechanism and species that communicate with weakly electric discharges (ED) (Hagedorn et al., 1990; Baron et al., 1994; Boyle et al., 2014; Kéver et al., 2021). These acoustic and electric signals are produced with the elastic spring apparatus which has been modified in the electrically communicating species (Boyle et al., 2014). As acoustic communication is the ancestral communication condition (Kéver et al., 2021), changes in the electrosensory circuit of the electro-communicating species can be, in part, attributed to the changes to a different communication modality. Thus, they are ideal models for studying the structural and neurochemical organization of different communication systems.

Across the different levels of sensory processing that are involved in perceiving social signals, the midbrain takes a special position in teleost fishes, being the key area where different sensory circuits interact and where motor actions are initiated. In the teleost midbrain, the torus semicircularis (TS), the homolog of the mammalian inferior colliculus, forms such an area. Its structural organization and connectivity have been studied in several species, including cyprinids (carps and minnows) (Echteler, 1984; Yamamoto and Ito, 2008; Yáñez et al., 2024), batrachoididae (toadfish) (Goodson and Bass, 2002), mormyrids (elephant fish) (Bell, 1981; Zeymer et al., 2018), gymnotiformes (knifefish) (Carr et al., 1981) and catfishes (Knudsen, 1976b,1976a,^1977^, ^1978^; Finger and Tong, 1984; Striedter, 1991). In general, acoustic input from the octaval nuclei in the hindbrain (namely the descending octaval nucleus DON, the secondary octaval nucleus SON and the medial auditory nucleus of the medulla MAN) reaches the central division of the TS (TSc), via the lateral lemniscus (LL) (Bass et al., 1994; Bass et al., 2000, 2001). The electrosensory subdivision of the TS, its lateral part (TSl), receives its input from the electro lateral line lobe (ELL) which is the primary electrosensory region (Finger and Tong, 1984). TSl is also indirectly connected to the ELL via the LL from the nucleus praeeminentialis (nPE). The nPE projects both directly and indirectly to the ELL and receives electrosensory information from the TSl (Finger and Tong, 1984; Bastian and Bratton, 1990; von der Emde and Bell, 1996). In catfish and other teleosts, tracer injections in the TSc and TSl have revealed direct or indirect projections to the anterior tuberal nucleus (AT) (Echteler, 1984; Striedter, 1991; Northcutt, 2006; Giassi et al., 2007) suggesting that auditory and electric information are processed in the diencephalic AT (Echteler, 1984; Striedter, 1991; Bass et al., 2000, 2001; Goodson and Bass, 2002; Petersen et al., 2013; Yáñez et al., 2024). In catfish, lateral line information also reaches AT through the mechanosensory subdivision of TS (TSv) (Striedter, 1991). Previous studies have shown that TSv receives its lateral line input coming directly from the primary sensory nucleus in the hindbrain through the LL (Striedter, 1991; Finger and Tong, 1984). The TS is thus a central hub of acoustic, lateral line, and electrosensory information and is therefore a prime candidate to investigate sensory adaptations to novel communication channels.

Besides the reported differences in connectivity in the toral subdivisions, other differences in organization have been reported in both TSl and TSc, including differences in patterns of calcium-binding protein (CBP) expression. Parvalbumin positive cells are broadly distributed across the whole extension of TS in the tench (*Tinca tinca*) (Crespo et al., 1999), similarly, calretinin and calbindin positive cells are present throughout the whole extension of the TS in sturgeons (Graña et al., 2012) and the gray mullet (Díaz-Regueira and Anadón, 2000). In some species, the distribution of CBPs is not as homogenous. In the TSl of mormyrids and the ventrolateral TS of zebrafish, for instance, a large number of calretinin positive cells are present. Other parts of the TS only occasionally contain scattered calretinin-expressing cells (Friedman and Kawasaki, 1997; Castro et al., 2006). The strong presence of calretinin in the electrosensory system has been proposed to facilitate the processing of electro-communicating signals (Friedman and Kawasaki, 1997). In some mammalian vocal communicating species, the distribution of CBPs in different auditory nuclei has been described and been proposed to contribute to the excitability of neurons (Arai et al., 1991; Vater and Braun, 1994; Lohmann and Friauf, 1996; Tardif et al., 2003; Alvarado et al., 2005; Clarkson et al., 2010), which may affect social communication processing. Due to the differences in CBP expression reported previously in different systems (see above), the question arises as to whether a change in communication modality is correlated with changes in neuronal processing. Such alterations might be indirectly observed by differences in protein expression, such as those previously reported for CBPs in the Torus (Friedman and Kawasaki, 1997). If adaptations to changes in communication modality are correlated with changes in neuronal processing, we might expect changes in CBP expression in the TSl of *Synodontis* that transitioned parts of their communication system from acoustic to electric.

Communication preference for conspecifics is well known in a number of fish species (e.g., McKibben and Bass, 1998; Schmidtke et al., 2013; Verzijden et al., 2010, Ward et al., 2020). In this study, we first tested whether individuals of *Synodontis grandiops* (SG) and *S. nigriventris* (SN) (two closely related catfish species) that employ different elastic spring related communication systems (vocal and electric, respectively) show a strong preference for conspecific signals in their preferred modality of communication. We then tested whether these behavioral differences can be correlated with differences in the structural and neurochemical organization of the brain regions processing and integrating those cues. We observed that the acoustic and electrosensory processing divisions of the TS revealed a conserved connectivity between the species employing different communication modalities. We identified a higher number of calbindin-immunoreactive cells in the TSc, while calretinin- and parvalbumin-positive cells were generally more abundant in the TSl of both tested species. These findings suggest that different CBPs have different importance in sensory systems, but do not indicate changes related to different communication modalities. Further experiments are needed to determine if evolutionary shifts in communication are accompanied by changes in neurochemical profiles and potentially by alterations in connectivity patterns in sensory processing regions in *Synodontis*.

## Materials and methods

### Husbandry

*S. grandiops* (SG) and *S. nigriventris* (SN) of undetermined sexes were obtained from commercial dealers (Tropicwater, Rodgau, Germany)^1^ and some of the SG were generously gifted from the Reichard laboratory. Fish were housed in the animal facility at the Institute of Biology at the University of Graz at a constant temperature of 25°C in 200 L aquariums with 12:12 h day and night cycle. Group size was limited to a maximum of 20 individuals. Water parameters were: conductivity 450–600 μS/cm, pH 7.2–7.5, hardness 14–18°dH. Animals were housed according to species requirements. Fish were fed daily with commercial dry and frozen fish food. The experiment was carried out following the ethical guidelines of the local animal care committee’s regulations.

All values reported in this study are average ± S.E.M.

### Two-choice preference test

To test behavioral preferences in the two *Synodontis* species, four different behavioral experiments were conducted. The behavioral experiments aimed to determine whether the tested species presented a preference toward conspecifics and if the absence of some sensory cues could disrupt that preference. All the experiments were conducted in aquaria of the same size (130 cm × 60 cm × 53 cm high), light (LED white eco chic, Tunze Aquarientechnik GmbH, Penzberg, Germany), and temperature conditions (25°C), except for the electric signal playback experiment which was conducted with the same light and temperature conditions but in a smaller sized aquarium (60 cm × 30 cm × 25 cm).

**FIGURE 1:**
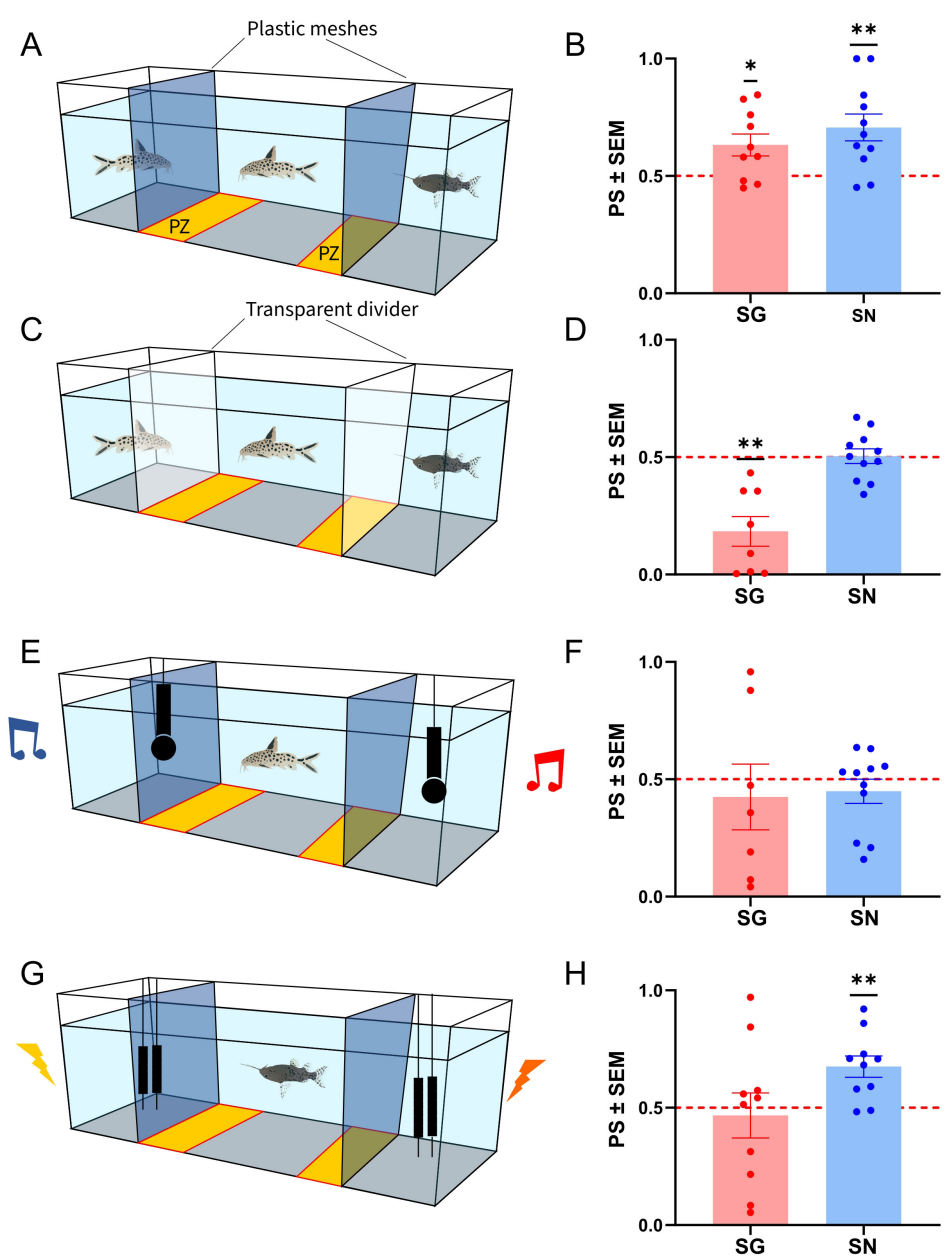
Schematic of the experimental setup and average preference scores(bar graphs, points indicate individual values) for the *no deprivation* experiment (**A,B**) [10 *Synodontis grandiops* (SG) and 11 *Synodontis nigriventris* (SN)], the *olfactory/electric cues deprived* experiment (**C**,**D**) (8 SG and 11 SN), the *auditory playback* (206 Hz vs. broadband white noise stimulus) experiment (7 SG and 11 SN) (**E,F**), and the *electric playback* (101 Hz vs. broadband frequency electric stimulus) experiment (**G,H**) (10 SG and 10 SN). PZ, preference zone; PS, preference score. Means and S.E.M. are shown. Asterisks (*) represent significant departures from the chance level of 0.5, marked by the dotted line (*p < 0.05; **p < 0.01).

In the first experimental set, the “*no deprivation*” experiment, 10 SG (mean fish standard length: 88 ± 6 mm, weight: 16.26 ± 2.44 g) and 12 SN (mean size: 51 ± 1 mm; weight: 4.35 ± 0.30 g) were used. A two-choice preference test was conducted in an environmentally controlled experimental room, dimly illuminated by a light source located at the top of the experimental room pointing to the ceiling. A transparent mesh was used to divide the aquarium into three partitions (15 cm width on the left and right side and 100 cm in the central compartment, Figure 1A). In the left and right aquarium compartments white plastic occluders were positioned in front of the plastic meshes before the experiment to prevent test animals to see these compartments. A camera (DFK 33UX287, 33U-Serie, The Imaging Source Europe GmbH, Bremen, Germany) recorded the experimental arena from above and images were saved via USB on a PC using ICCapture software (The Imaging Source Europe GmbH, Bremen, Germany).

Fish were randomly assigned to the experiments. Each test subject was habituated overnight to the experimental aquarium. On the day of the test, one conspecific and one heterospecific were positioned in the test areas located behind the meshes and the occluders and were thus not visually detectable to the test subject. The fish that were used as “stimuli” were not housed in the same tanks as the test subjects and had not encountered these fishes previously. The two white occluders in front of the test compartments were removed before the session started. Between trials, the position of the conspecific was altered to avoid side preferences in the choice compartments. At the beginning of the recording session, the partitions in the tank were removed to allow the test subject to see both animals located in the choice compartments during the experimental sessions. The fish was video-recorded from above at five frames per second for 1 h. After the test we transferred the test subjects to other tanks to avoid testing the same individual repeated times (within one experimental set).

In the “*olfactory/electric cues deprived*” experiment, two transparent polyplex dividers were glued into the tank instead of the transparent mesh to exclude olfactory (and electroreceptive) cues coming from the fish on the test compartments Figure 1C. Behavioral preference was recorded as detailed above in the experiments in which a plastic mesh was used to separate the tank into three compartments. In this set of experiment 12 SG (mean size: 66 ± 1 mm, weight: 5.27 ± 0.31 g) and 12 SN (mean size: 86 ± 1 mm, weight: 4.5 ± 0.87 g) were used.

In the “*auditory playback*” experiment, a loudspeaker (UW 30 Lubell labs, Ohio, United States) was positioned in each test compartment (using the tank with the compartments separated by plastic mesh, Figure 1E). The speaker in one compartment presented a 206 Hz sine tone [similar to the fundamental frequency of swim bladder tonal vocalizations produced by SG (Kéver et al., 2020)]. We exposed both species to this sound as SN does not generate swim bladder associated sounds. The speaker in the other compartment presented a broadband sound (Frequency range: 100–3,000 Hz. Due to the non-linearity of the speakers, and the difficulty of sound reverberation in small aquaria, sound amplitudes were not equally distributed. Both sounds had a duration of 110 ms and a ramp of 5% to avoid onset effects. Each sound was presented repetitively for 1 h with inter-sound pulses of 2 s. Sounds were presented in alternation, with a 1 s delay between the two sound sources. To adjust the sound amplitude, we recorded sounds form a group of 5 SG, extracted the relative amplitude and adjusted our artificial sounds to match these amplitudes in the respective preference zones. The speakers were each driven by an amplifier (NAD ELECTRONICS LTD, 214 stereo power amplifier; Pickering, Canada) connected to a laptop computer. Behavioral preference was recorded in 12 SG (mean size: 62 ± 2 mm, weight: 4.7 ± 0.33 g) and 12 SN (mean size: 44 ± 1 mm, weight: 2.46 ± 0.11 g).

In the “*electrical playback*” experiment, a pair of custom-made silver electrodes (anode and cathode, positioned 2.5 cm from each other - oriented parallel to the length axis of the tank) were positioned in each test compartment Figure 1G. Test compartments were separated from the main compartment by plastic mesh to prevent the fish to enter these compartments. Electrode pairs on one side presented a previously recorded SN electric discharge, whose characteristics were similar to those previously reported by Kéver et al. (2020): 29 pulses with pulse intervals of ∼110 Hz with amplitude modulation. We only presented this SN communication signal, as SG does not produce electric discharges associated with the elastic spring mechanism. In the other compartment a broadband electric signal was played. Both signals had a duration of 265 ms with a 2.5 s interevent pause looped for 1 h. Signals were play in alternation with an initial delay of 1 s. The peak amplitude of each signal was set to 300 μV and adjusted prior to each experiment. Signals were generated digitally and fed through an AD converter (CED 2701, Serial# p2073, United Kingdom) to an attenuator (PA5 programmable attenuator, TDT) and a linear stimulus isolator (A395R, WPI, INC., Sarasota, United States) to the electrodes in the tank. The transparent mesh was used to divide the aquarium into three partitions (12 cm width on the left and right side and 36 cm in the central compartment). Behavioral preference was recorded in 12 SG (mean size: 55 ± 2 mm, weight: 3.61 ± 0.30 g) and 12 SN (mean size: 44 ± 1 mm, weight: 2.79 ± 0.22 g).

#### Preference score analysis

Video recordings of the preference tests were analyzed using DeepLabCut (Version 2.2.3) (Mathis et al., 2018; Nath et al., 2019). To track the position of the test animal within the tank, we trained DeepLabCut with 20 videos in which we labeled 20 frames each for SG and 12 videos with 40 frames each for SN. We assigned 1 label on the head of each fish. This label was subsequently used to identify the x/y coordinates (i. e., the location) of the fish in the experimental arena. We did not determine the orientation of the fish. To test if the fish showed a preference for the conspecifics or the conspecific cues, we used a custom written Matlab script (Matlab v. R2020b, The MathWorks, Inc., Nattick, United States), that scored the position of the fish for each second of the video recording. We divided the central compartment into three analysis segments: a middle (50 cm) and two end segments, referred to as preference zones (each 25 cm long; Figure 1A). To calculate the preference score, the overall time spent in the choice compartment of the conspecific was divided by the overall time spent in both preference zones. The preference score ranges from 0 to 1, with a value of 1 indicating an absolute preference for the conspecific (signal) and a value of 0 indicating an absolute preference for the heterospecific (noise signal). Animals that didn’t explore the preference zones in the first 30 min of the test were excluded from further analysis (*n* = 1 SN). We applied the IQR detection method (Chambers, 2018) to check for outliers in each species and those were excluded (“*no deprivation*” experiment: 1 SN, 0 SG; “*olfactory/electric cues deprived*” experiment: 1 SN, 0 SG; “*auditory*” experiment: 0 SN, 0 SG; “*electric*” experiment: 1 SN, 0 SG) from the final sample size in which we analyzed the preference scores. To analyze the performance of all fish, a one sample, two-tailed *t*-test was used to reveal significant differences from chance level (i.e., a preference score of 0.5). After testing the normality of our data (Shapiro-Wilk test), a one sample *t*-test or a one sample Wilcoxon aligned ranks test was used to test for differences in the preference score between the two species. Statistical analysis of the behavioral data was performed with the software GraphPad (Version 9.5.0).

### *In vitro* tracing of the toral subdivisions

To test for differences in toral projections we used 10 SN for *in vitro* tracing experiments (mean length: 42 ± 2 mm, mean weight: 1.96 ± 0.18 g) and 11 SG (43 ± 1 mm, 1.59 ± 0.12 g). Animals were first deeply anesthetized (0.05% MS222 in tank water) and subsequently decapitated. The skull was quickly removed to expose the brain in ice-cold Ringers’ solution (134 mM NaCl, 2.9 mM KCl, 2.1 mM CaCl_2_, 1.2 mM MgCl_2_, 10 mM Hepes, 10 mM Glucose, pH 7.8 adjusted with NaOH) for freshwater teleost fish (Kyriakatos et al., 2011). The dorsal part of the TeO of the left hemisphere was removed to expose the TS. Glass micropipettes (∼2–3 μm tips, Science products GmbH, Taunus, Germany) containing 10% neurobiotin (Vector Laboratories, Inc, SP-1120, Burlingame, CA, United States) in 0.5 M potassium acetate in deionized water were positioned into the TS using a micromanipulator (Narishige, Model: M-3333, Science products, GmbH, Austria). Injections were performed either in the lateral (TSl) or the central nucleus (TSc) of the TS. A pulse generator (GRASS Instruments, Model: SD9D, United States) connected to a stimulus isolator (World Precision Instruments, Model: A365R, Sarasota, United States) was used for iontophoresis. Current pulses (10 μA with a stimulus duration of 200 ms at a frequency of ∼2 Hz) were continuously applied for 15 min.

After the iontophoresis, brains were transferred for ∼10 h at 5–7°C to a continuously carbogenated Ringers’ solution (Berg et al., 2018) to allow the neurobiotin to travel. Brains were then fixated overnight in 4% PFA in 0.1M PB at 4°C. Brains were embedded in 4% agar, sectioned in the coronal plane at 75 μm on a vibratome (Campden instruments, Model: 7000SMZ -0268, Loughborough, United Kingdom), and directly mounted onto gelatine-coated microscope slides. Sections were washed in 0.1 M PBS and incubated for 2 h in a 1:500 streptavidin 647 solution (Invitrogen, S21374, Life Technologies, Carlsbad, CA, United States) in 0.1 M PBS with 0.03% Triton-X. Slides were then cover-slipped with mounting medium (ROTH, ROTI Mount FluoCare with DAPI, Karlsruhe, Germany), and nail polish was applied on the slide borders to prevent the mounting medium from drying. The sections were examined with an epifluorescence microscope (ZEISS, imager.M2, Jena, Germany).

### Expression of selected calcium binding proteins in the torus semicircularis

To identify the pattern of expression of different calcium binding proteins (CBPs) inside the TS, immunohistochemical labeling was performed on 12 SG (mean size: 50 ± 2 mm, weight: 2.97 ± 0.35 g) and 11 SN (mean size: 50 ± 2 mm, weight: 3.81 ± 0.41 g). Six SG and 6 SN were used for calretinin (SG mean size: 48 ± 2 mm; mean weight: 2.49 ± 0.27 g; SN mean size: 50 ± 3 mm; mean weight: 4.22 ± 0.57 g) and for parvalbumin (SG: 48 ± 2 mm; 2.67 ± 0.27 g; SN: 54 ± 2 mm; 4.46 ± 0.50 g), while 7 SG and 7 SN were used for calbindin (SG: 49 ± 3 mm; 3.08 ± 0.58 g; SN: 48 ± 3 mm; 3.75 ± 0.60 g). Fish were euthanized with 0.05% MS-222 (Pharmaq. Ltd, Hampshire, United Kingdom) in tank water and subsequently perfused with ice-cold Ringers’ solution and 4% PFA in 0.1 M PB at 4°C. After post-fixation overnight, the brains were removed from the skulls and stored in 0.1 M PBS for 1–7 days at 4°C. The brains were cryoprotected in 30% sucrose in 0.1 M PBS overnight at 4°C before being sectioned in the coronal plane in three series at 25 μm on a cryostat (Leica, Model: CM3000- 1-, Wetzlar, Germany) and mounted directly onto gelatine-coated slides. Slides were dried under a fume hood overnight at room temperature and stored at −20°C until processing. Individual series were used to stain different CBPs.

#### Immunohistochemical staining

Sections were first rehydrated and washed in 0.1 M PBS for 3 × 5 min. Unspecific binding sites were blocked with 5% normal donkey serum (0171-000-121, Jackson Immunoresearch Laboratories, INC., Cambridgeshire, United Kingdom) in 0.1M PBS containing triton-X (0.3%) for 1 h at room temperature and then washed in PBS for 3 × 5 min. Different series were incubated with following different antibodies: To detect calretinin we used an anti-calretinin antibody (1:1000; SWANT; Cat# CR 7697, RRID:AB_2619710, Lot# 1893-0114; made in rabbit; Burgdorf, Switzerland). This calretinin antibody has been used in diverse fish species such as the blind cavefish (*Astyanax mexicanus*), the giant Danio (*Devario aequipinnatus*) and the paradise fish (*Macroposus opercularis*) (Folgueira and Clarke, 2024), and in zebrafish (*Danio rerio*) (Porter and Mueller, 2020, Turner et al., 2022, Berg et al., 2018). For calbindin we used an anti-calbindin antibody (1:1000; SWANT; Cat# cb38a, RRID:AB_3107026, Lot# 9.03; made in rabbit; Burgdorf, Switzerland) that has been used in diverse fish species such as in the saddled bichir (*Polypterus endlicherii*) and the snake fish (*Erpetoichthys calabaricus*) (Lozano et al., 2025), and the shorthorn sculpin (*Myoxocephalus scorpius)* (Olsson, 2011). The antibody has also been tested with a western blot by the manufacturer in zebrafish (*Danio rerio*) (see manufacturer homepage). For parvalbumin we used an anti-parvalbumin antibody (1:1000; SWANT; Cat# PV27, RRID:AB_2631173, Lot# 2014; made in rabbit; Burgdorf, Switzerland) has been used in the turbot (*Psetta maxima*) (De Miguel and Álvarez-Otero, 2017) and in zebrafish (*Danio rerio*) (Berg et al., 2018; Kenney et al., 2021). Slides were incubated for 48 h at 4°C in 2% normal donkey in 1X PBS-triton (0.3%) solution. After three washes with PBS, slides were incubated for 2 h at room temperature with secondary antibodies (Alexa Fluor 546; 1:500; Invitrogen, A10040, made in donkey, Life Technologies, Carlsbad, CA, United States) or Alexa 488 conjugated anti-rabbit (1:500; Invitrogen, A21206, made in donkey, Life Technologies, Carlsbad, CA, United States) in PBS-triton with 5% normal donkey blocking solution. Slides were quickly rinsed in double distilled water, mounting medium (ROTH, ROTI Mount FluoCare with DAPI; Karlsruhe, Germany) applied and slides cover-slipped. The slides were sealed with nail polish to prevent the mounting medium from drying. We tested for non-specific binding for all primary antibodies by carrying out the staining protocols above but omitting the primary antibodies. All tests revealed no staining.

### Quantification of calcium-binding protein distribution

Immunoreactivity of the different CBPs was analyzed in the central and lateral subdivisions of the TS. Brain sections were examined with a Zeiss Axio Imager2 microscope and a digital camera (Zeiss Axiocam 305 mono, ZEISS, Jena, Germany). Counting of CBPs-ir cells was performed manually using the cell counter in Fiji (ImageJ, v. 1.53f51, National institutes of health, Wayne Rasband, United States) blind to the experimental conditions. The TSl and TSc were identified based on the landmarks in the DAPI stainings. The quantification of CBP immunoreactive cells was performed in five sections of each fish for both hemispheres. These sections were selected across the rostro-caudal extent of the TS in both species. The selected sections were those with the observed highest number of labeled cells in both toral subdivisions, which were then counted after manual tracing of the borders of the two toral subdivisions. We measured the cell densities of each toral region in both species. These cell densities were averaged and standardized to cells/mm^2^. Since there were no visually apparent differences between the hemispheres in any brain regions, we randomly selected a hemisphere per fish from which we obtained the density value. The density values were normalized by the DAPI intensity to account for any possible differences in the overall number of cells present in the torus of SG and SN. The resulting individual values were considered representative of the overall CBP in each brain region and were used for further statistical analysis.

To test for potential differences in the CBPs cell densities between SG and SN, we used linear mixed effect models using the *lme* function from the *nlme* package (Mangiafico, 2016) in the R programming language (R Core Team, 2023). Models coded subject as a random intercept to account for repeated measurements and residuals were weighted following an exponential kernel (“VarExp”) proportional to the fitted value. For *post hoc* analyses, parametric bootstrapping was performed, based on random resampling of the model parameter distributions returned by *nlme* (Mangiafico, 2016).

### Volumetric analysis of TS on histological sections

To test for potential volumetric shifts in the TS, we delimited the TS and its two TS subdivisions using DAPI staining in sections taken from immunohistochemical experiments (SG: mean size: 48 ± 2 mm; weight: 2.49 ± 0.27 g; N = 6; SN: mean size: 50 ± 3 mm; mean weight: 4.22 ± 0.57 g; *N* = 6). We sampled the TS every 75 μm (as three series were cut) and measured the area of the two TS subdivisions (TSc and TSl) in one hemisphere. The mean area of each toral subdivision was normalized by the overall area of the TS for each fish. These values were then multiplied by a factor of 75 (25 μm section thickness, but only every fourth section was analyzed) to obtain the relative volume of these brain nuclei. To test for volumetric shifts, we used two-way ANOVA after checking the normality of our data (Shapiro-Wilk test of normality). Multiple comparisons *t*-test was used for *post hoc* analysis. We randomly selected five slices in each animal to be remeasured twice, blind to previous results and species identity as a control.

## Results

### Behavioral preference tests

To test whether *Synodontis* show a preference toward conspecifics and to different communication modalities, we exposed individuals of SG and SN to a series of two-choice tests. In the first test, (hereafter referred to as *no deprivation* test), subjects were exposed to con- and hetero specifics without limitations to visual, acoustic, electric, or olfactory cues. Social preference was calculated by the time spent in a preference zone next to the conspecific (see section “Materials and methods”). SN and SG showed an average preference score of 0.707 ± 0.057 and 0.632 ± 0.048 respectively. A one-sample, two-tailed *t*-test (after checking for normality) revealed a significant conspecific preference in both species (Normality test: Shapiro-Wilk’s method in SG: w = 0.921, *p* = 0.366; in SN: w = 0.949, *p* = 0.635; *t*-test in SG: t_(9)_ = 2.830, *p* = 0.020; in SN: t_(10)_ = 3.624, *p* = 0.005, Figure 1B).

We next tested whether social preference depended on the presence or absence of olfactory and electrical cues (*olfactory/electric cues deprived* experiment). We repeated the same experiment as the *no deprivation condition*, however, instead of using a plastic mesh to separate the compartments, we used plastic dividers that were glued into the tank. This prevented olfactory and electric cues of the lateral compartments to reach the tested subject in the middle compartment. Both species showed an absence of attraction to conspecifics (Shapiro-Wilk in SN: w = 0.965, *p* = 0.837; *t*-test in SN: t_(10)_ = 0.124, *p* = 0.904, Figure 1D; average preference score SN 0.504 ± 0.031 and SG 0.184 ± 0.063). In SG, the absence of olfactory and electric cues resulted in an avoidance of conspecifics (Shapiro-Wilk in SG: w = 0.841, *p* = 0.837; *t*-test in SG: t_(7)_ = 4.995, *p* = 0.002).

To test if *Synodontis* showed a preference toward auditory communication signals, we presented a two-choice scenario using two different acoustic cues (*auditory playback* experiment): One at the fundamental frequency of SG tonal swim bladder vocalizations on one side of the test arena, and one with a broad band noise stimulus of equal duration on the other side. SN and SG showed average preference scores of 0.449 ± 0.052 and 0.425 ± 0.140, respectively. After testing for normality, a one-sample Wilcoxon signed ranks test for SN and a one-sample two-tailed *t*-test for SG revealed no preference for either cue in either species (Normality test: Shapiro-Wilk’s method in SN: w = 0.793, *p* = 0.008; in SG: w = 0.922, *p* = 0.487; SN: W = −8.000, *p* = 0.765; SG: t_(6)_ = 0.538, *p* = 0.610, Figure 1F).

We also tested for a potential preference toward different electric stimuli, by presenting fish with a stimulus with the same waveform and frequency as those emitted by SN during social interactions and as alternative, a broadband noise electric stimulus. SN and SG showed average preference scores of 0.675 ± 0.046 and 0.467 ± 0.096, respectively. A one-sample two-tailed *t*-test revealed a preference for conspecific electric cues in SN (Normality test: Shapiro-Wilk’s method in SN: w = 0.945, *p* = 0.611; t_(9)_ = 3.837, *p* = 0.004, Figure 1H) but no preference for SG (Normality test: Shapiro-Wilk’s method in SG: w = 0.868, *p* = 0.095; t_(9)_ = 0.345, *p* = 0.738).

### Volumetric shifts in histological sections

**FIGURE 2:**
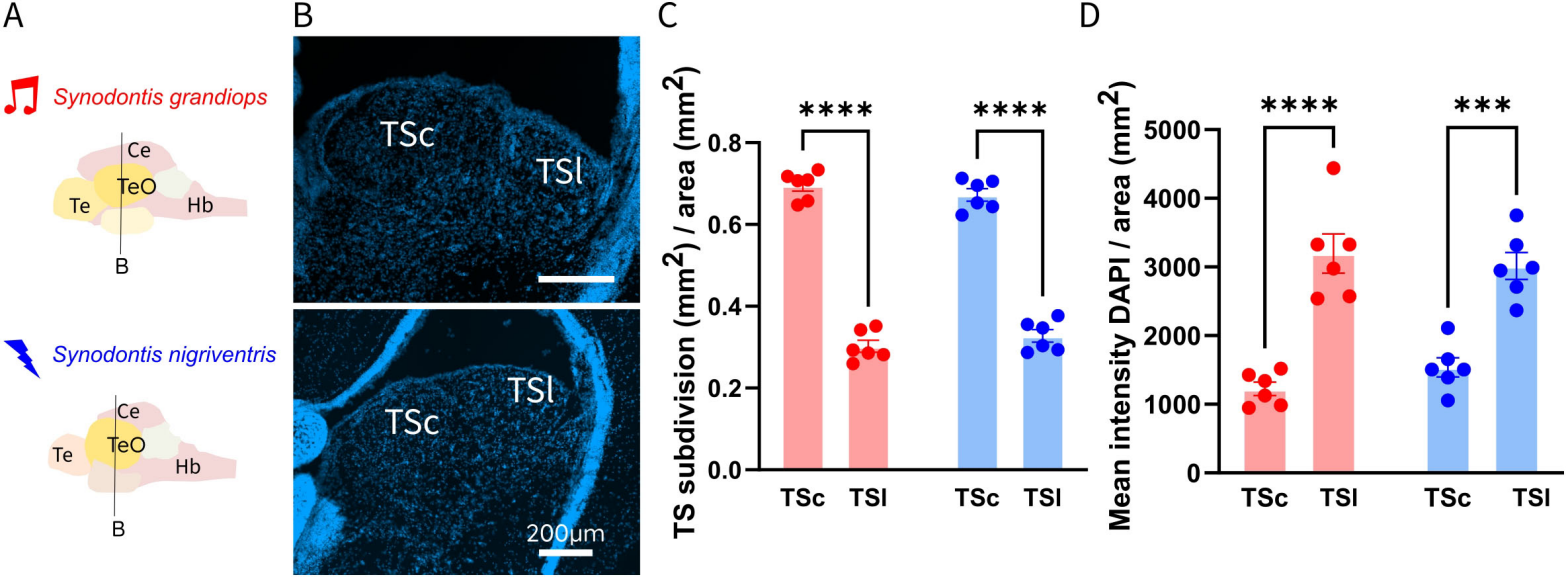
**(A)** Schematic representation of brains of *Synodontis grandiops* (SG) and *Synodontis nigriventris* (SN). Black lines indicate the location of the coronal sections depicting the torus semicircularis (TS) shown in **(B)**. **(B)** Coronal sections at the level of TS in SG (upper part) and SN (lower part). **(C)** Bar graphs depicting the averaged central (TSc) and lateral torus (TSl) size (normalized to respective TS area) for SG (*N = 6*) and SN (*N = 6*). **(D)** Normalized DAPI intensity values in the TS subdivision of both species. Te, telencephalon; Ce, cerebellum; Hb, hindbrain. Bar plots indicate mean S.E.M. (***P < 0.001; ****p < 0.0001).

To test whether the behavioral preference of SN to electrical social signals is reflected in the nervous system, we investigated the torus semicircularis (TS), a central hub of sensory information processing. We therefore examined the relationship between the electrosensory TSl and the acoustic TSc, by measuring their volumes (normalized to the volumes of the respective TS) in histological sections (Figures 2A, B). The toral subdivisions showed a significant effect of area (*p* < 0.0001), but no effect of species (*p* = 0.147) or interaction between species and areas (*p* = 0.278), as tested via a two-way ANOVA (Normality test: Shapiro-Wilk’s method in SG TSl: w = 0.889, *p* = 0.315; TSc: w = 0.877, *p* = 0.254; in SN: TSl: w = 0.899, *p* = 0.365; TSc: w = 0.899, *p* = 0.365). Our *post hoc* analysis revealed a significantly bigger TSc (auditory area of the TS) in both species (SG: 0.696 ± 0.014; SN: 0.673 ± 0.015; adjusted *p* < 0.0001; in SG and SN; Figure 2C), in comparison to their respective TSl (electrosensory area of the TS).

To test if an increase in cell number or cell size accompanied the differences in volume reported, we measured the intensity of DAPI staining in the two toral nuclei (Figure 2D). This measurement, does not differentiate between an increase in nuclei size or an increase in the amount of nuclei. The comparison of the DAPI intensity in the TSl and TSc revealed a significant effect of area (TSc and TSl) (*p* < 0.001) but no interaction (*p* = 0.209) between species (SG and SN) or an effect of species (*p* = 0.754). These results indicate a difference between the TSl and the TSc in both species, but no variation between the species. Our *post hoc* multiple comparison test of the two toral subdivisions indicated a higher DAPI intensity in the electrosensory subdivision of both species (3197.703 ± 285.619; adjusted *p* < 0.001 in SG; 3014.209 ± 195.739; adjusted *p* < 0.001 in SN).

### Toral connectivity in species with different communication modalities

To test for potential differences in connectivity patterns of the electrosensory and acoustic networks that might have arisen during the change in communication modality, we performed tract tracing experiments for both subdivisions of the TS.

#### TSl connectivity patterns

**FIGURE 3:**
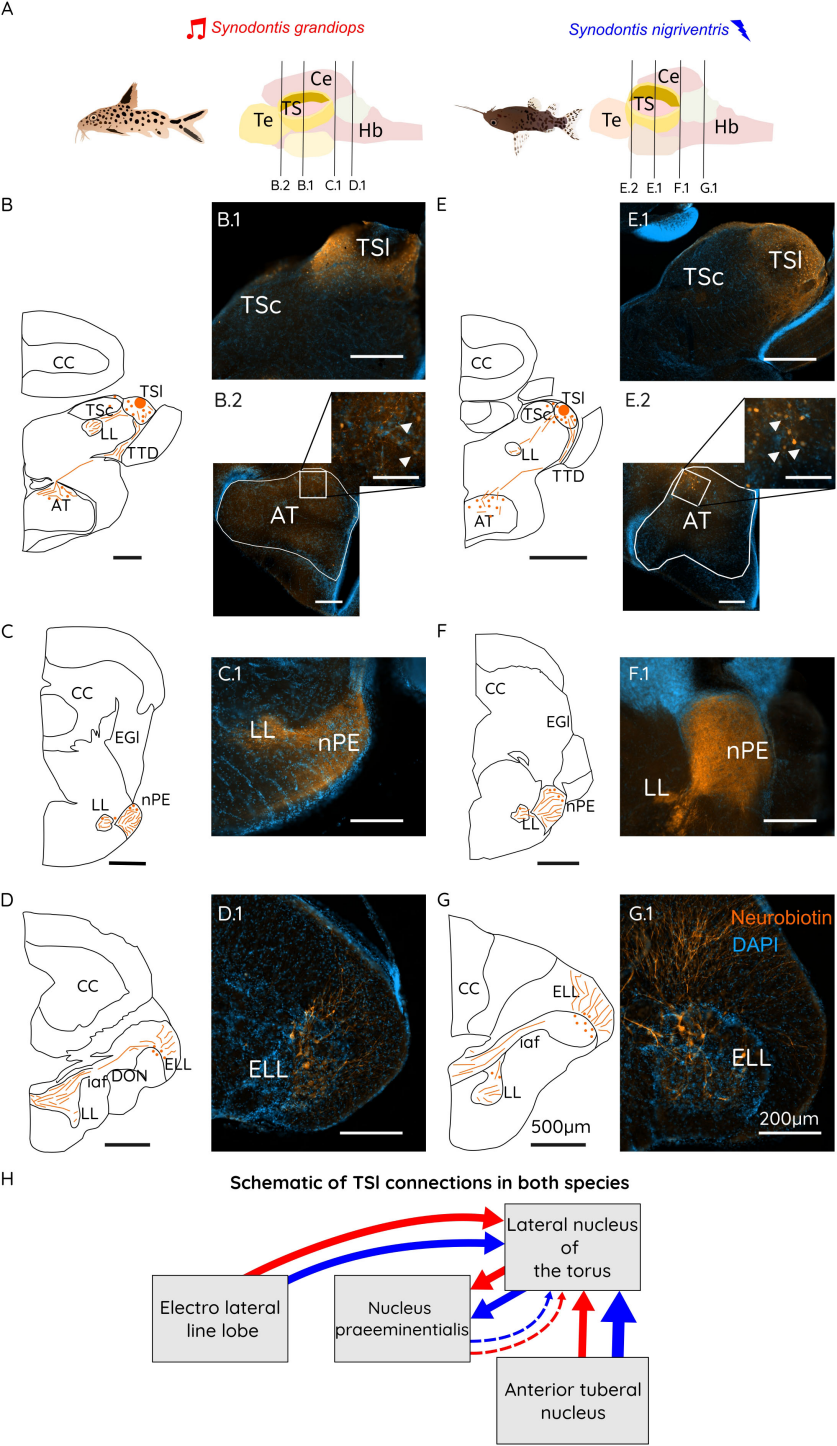
**(A)** Schematic representations of a *Synodontis grandiops* (SG) and *Synodontis nigriventris* (SN) fish and brain. Black lines represent the different cutting planes for sections shown below. Schematic drawings of brain sections containing the TS **(B,E)**, the nPE **(C,F)** and the ELL **(D,G)**. Photographs of neurobiotin stained sections for the TS region taken on an epifluorescence microscope (B.1, E.1), the AT (B.2, E.2), the nPE (C.1, F.1) and the ELLL (D.1, G.1). **(H)** Schematic diagram of the connections of the lateral toral nucleus (TSl) in SG (red arrows) and SN (blue arrows). Arrow thickness represents the strength of the connection between the indicated brain regions. Scale bars in the schematizations represent 500 μm in all drawings, 100 μm in the inserts and 200 μm in all stained sections. Te, telencephalon; TS, torus semicircularis; Ce, cerebellum; Hb, hindbrain; CC, corpus cerebelli; DP, dorsal posterior thalamic nucleus; LL, lateral lemniscus; AT, anterior tuberal nucleus; TSc, central nucleus of the torus semicircularis; TSl, lateral nucleus of the torus semicircularis; TTD, torodiencephalic tract; EGl, lateral eminentia granularis; nPE, nucleus praeeminentialis; DON, descending octaval nucleus; iaf, internal arcuate fibers; ELL, electro lateral line lobe.

Following injections of neurobiotin in TSl (Figure 3), we found dense labeling of fibers, putative terminals and cells on the injection side (Figure 3: B.1 in SG and E.1 in SN). Only few cells were found in the ipsilateral TSc. These cells have previously been described in other teleost fish (Midshipman, *Porichthys notatus*) (Weeg and Bass, 2000). Labeled fibers leave the TSl through its medial part and projected to the lateral lemniscus (LL) or through the lateral part of the nucleus following the toro-diencephalic tract (TTD). On the contralateral side, we observed fibers and cells only in the TSl, in accordance with previous reports from other fish (bullhead catfish, *Ictalurus nebulosus*; midshipman, *Porichthys notatus*) (Striedter, 1991; Weeg and Bass, 2000). Labeling was also found in the nPE and labeled the ipsi- and the contralateral side: At the level of the ipsilateral nPE we observed a dense fiber system, and putative terminals distributed in its dorsal and ventral subdivision (Figure 3: C.1 for SG and F.1 for SN) with sparse cells found in the dorsal-anterior part of this region. In contrast, in the contralateral nPE, only the dorsal subdivision was densely labeled with fibers and putative terminals. In the LL, fibers traveled further caudally and exited at the level of the internal arcuate fibers (iaf). The larger portion of the axons left the ipsilateral LL to enter the iaf and cross to the contralateral side, while some continued in the ipsilateral iaf. These fibers enter the ELL through its medial edge (Figures 3D, G; schemes in SG and SN). At the level of the ELL (Figure 3: D.1 for SG and G.1 for SN), we found retrogradely labeled crest cells bilaterally (Figure 3: D.1 for SG and G.1 for SN). Stronger contralateral labeling of crest cells was found in the ELL.

In the diencephalon, TTD fibers primarily terminated in the dorsal portion of the anterior tuberal nucleus (AT), with sparse branching into most of the nucleus in SN, while remaining predominantly dorsal in SG. In SN, neurobiotin-labeled cells were sparsely distributed throughout AT, whereas in SG, few cells were labeled. The observed sparsely labeled AT cells in SN indicate stronger connectivity to this region in the electro-communicating SN compared to the vocal SG (see Figure 3: B.2 for SG and E.2 for SN).

#### TSc connectivity patterns

**FIGURE 4:**
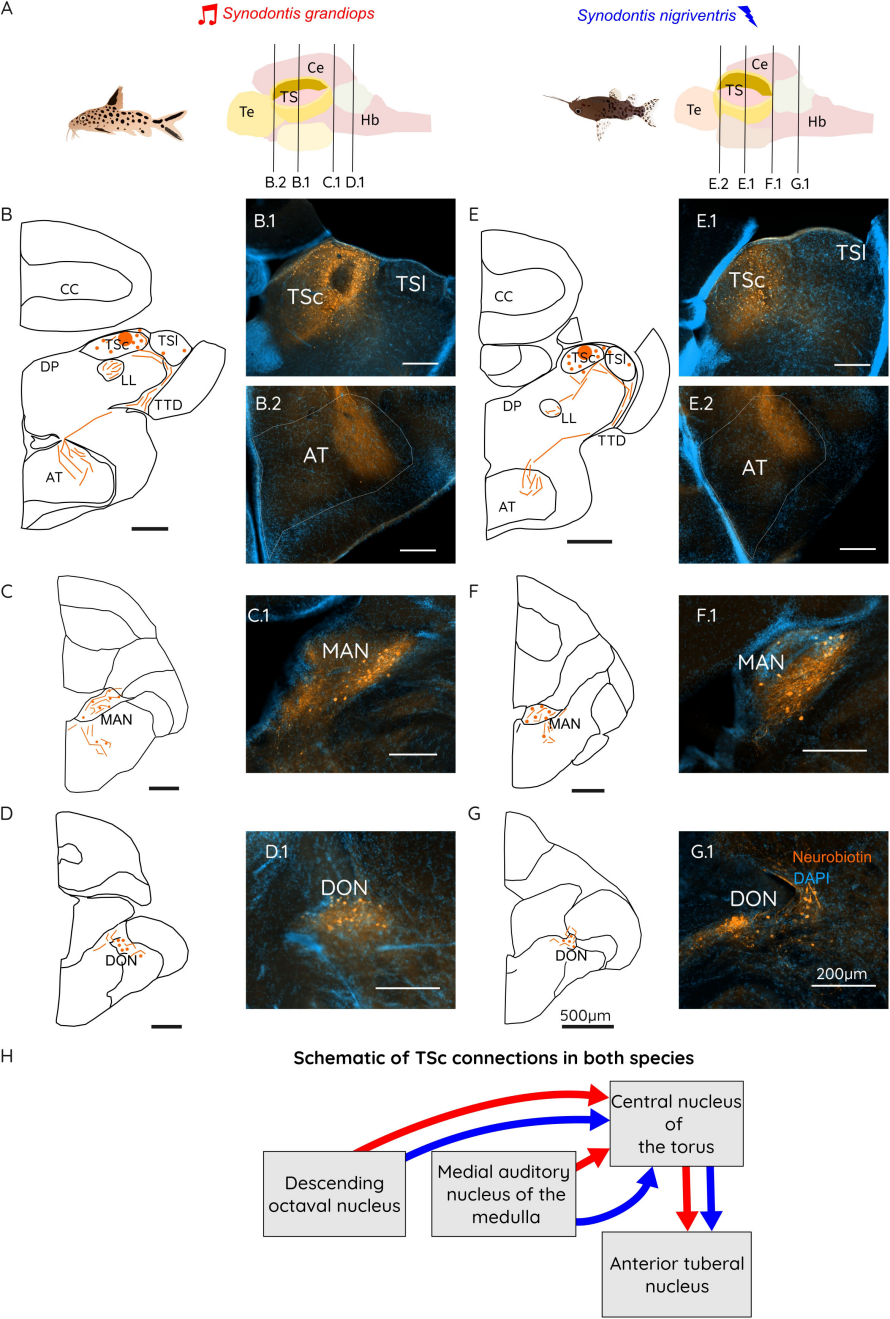
**(A)** Schematic representation of a *Synodontis grandiops* (SG) and *Synodontis nigriventris* (SN) brain and fish. Black lines represent the different cutting planes for sections shown below. Schematic drawings of brain sections containing the TS **(B,E)**, the MAN **(C,F)** and the DON **(D,G)**. Photographs of neurobiotin stained sections for the TS region (B.1, E.1), the AT (B.2, E.2), the MAN (C.1, F.1) and the DON (D.1, G.1). **(H)** Schematic diagram of the connections of the central toral nucleus (TSc) in SG (red arrows) and SN (blue arrows). Arrow thickness represents the strength of the connection between the indicated brain regions. Scale bars in the schematizations represent 500 μm in all drawings and 200 μm in all stained sections. Te, telencephalon; TS, torus semicircularis; Ce, cerebellum; Hb, hindbrain; CC, corpus cerebelli; DP, dorsal posterior thalamic nucleus; LL, lateral lemniscus; AT, anterior tuberal nucleus; TSc, central nucleus of the torus semicircularis; TSl, lateral nucleus of the torus semicircularis; TTD, torodiencephalic tract; MAN, medial auditory nucleus of the medulla; DON, descending octaval nucleus.

Tracer injection in the TSc revealed a conserved teleost connectivity of acoustic processing (Figure 4). Fibers labeled in TS were found connecting through the LL to the MAN (Figures 4C, F: C.1 for SG and F.1 for SN), and the DON, especially in its dorsal part (Figures 4D, G: D.1 for SG and G.1 for SN). In MAN, cells appeared to have been labeled mostly ipsilaterally with a roughly equal number of fibers on both sides. In the DON, cells were observed bilaterally (Figures 4D, G; D.1 for SG and G.1 in SN) although most cells were found ipsilateral to the injection site.

In the diencephalon, fibers arising from the TTD terminated in the external part of the central posterior nucleus of the thalamus (auditory thalamus) or in the lateral division of the anterior tuberal nucleus (AT) of the hypothalamus. The input to AT from TSc appears to describe a lateral subdivision of this nucleus (Figures 4B, E: B.2 for SG and E.2 for SN), in contrast to the sparse fibers and cells located in this region after injections in the electro-sensory subdivision of the torus.

### Distribution of calcium-binding proteins in the toral subdivisions

Having identified differences in the volume and cell densities of the different toral divisions, we next investigated whether cells in the TS showed different identities. We therefore performed immunohistochemical experiments to test for differential expression of three CBPs (calretinin, calbindin, and parvalbumin). All three were expressed in the TS, yet at various amounts and in different combinations in the two toral nuclei investigated.

**FIGURE 5:**
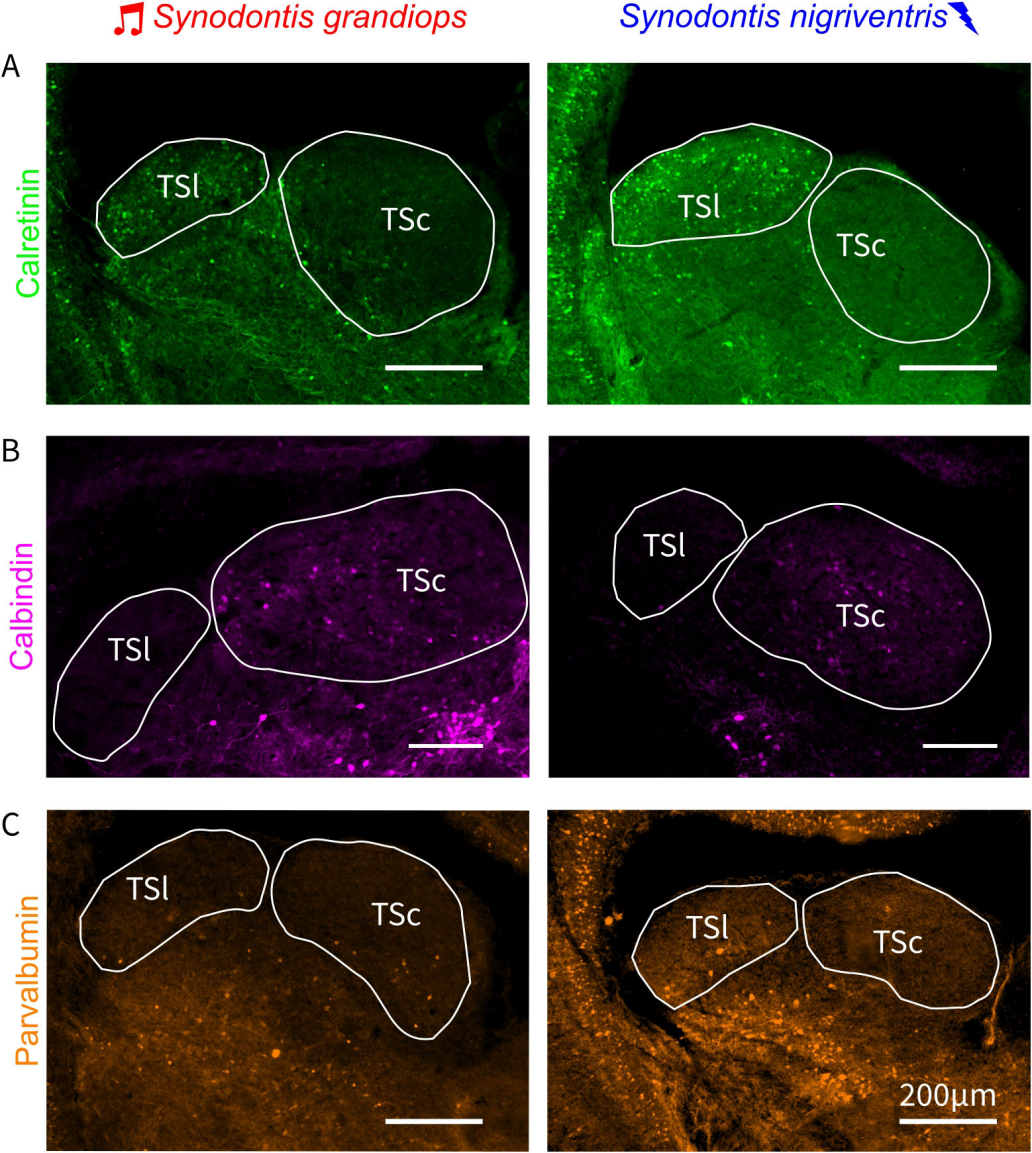
Cell densities of CBP-ir cells across the lateral and central torus semicircularis. Photographs of torus semicircularis (TS) sections after immunohistochemically processing for calretinin **(A)**, calbindin **(B)**, and parvalbumin **(C)** for *Synodontis grandiops* (SG – left panels) and *Synodontis nigriventris* (SN, right panels). TSc, central nucleus of the torus semicircularis; TSl, lateral nucleus of the torus semicircularis.

Calretinin immunoreactive (CR-ir) cells were abundantly found across the rostro-caudal extent of the TSl in both SG and SN (Figure 5A and Supplementary Figure 1). Interestingly, only a few CR-ir cells were found in the TSc of both species. While only some CR-ir fibers were present in the rostral part of TSc, abundant fibers and scattered cells were visible in the ventral nucleus of TS (TSv) as shown in Supplementary Figure 1 A1, A2.

Calbindin immunoreactivity (CB-ir) was prominently found in the TSc in both SG and SN. Only a few CB-ir cells were found in the TSl of either species (Figure 5B and Supplementary Figure 1). A more abundant population of cells and fibers was observed in the TSv (see Supplementary Figure 1 B1, B2).

Parvalbumin immunoreactive (PV-ir) cells were rarer than the two other CBP and were scattered in both toral nuclei in both species (Figure 5C and Supplementary Figure 1), with more cells present in the TSl of SN (see analysis below). Putative PV-ir terminals (indicated by possible varicosities in Supplementary Figure 1 C1, C2) were located in the TSc and TSl of both species. Finally, PV-ir cells and fibers were abundantly found in the TSv of both species.

We first quantified the densities of CBP-ir cells (all CBPs taken together) in the TSl and TSc of both species, and subsequently used linear mixed effect models to statistically test for changes in CBP expression. This revealed a significant interaction between species and area (*p* = 0.043); however, we did not detect an effect of area (*p* = 0.071) or an effect of species (*p* = 0.110). A *post hoc* bootstrap analysis revealed that significantly higher CBPs-ir cell densities were present in the TSl of SN in comparison to the TSl of SG (log mean cell densities (number of ir-cells/mm^2^): TSl SN: 4.475 ± 0.473; TSl SG: 3.614 ± 0.560; *p* = 0.003). In SN, a difference in CBPs-ir cell densities was present between TSl and TSc (TSc SN: 1.988 ± 0.158; *p* = 0.038). However, there was no difference in the number of CBPs-ir cells between the lateral and the central toral nuclei of SG (TSc SG: 1.788 ± 0.152; *p* = 0.347). Finally, no differences were found between the TSc of SG and SN (*p* = 0.300; for a complete list of the *post hoc* results see Table 1) restricting the differences found to the electrosensory subdivision of the TS.

**TABLE 1:** *Post hoc* bootstrap analysis of calcium binding proteinimmunoreactive cells in the central and lateral toral subdivision between *Synodontis grandiops* (SG) and *Synodontis nigriventris* (SN).

*Post hoc* bootstrap analysis of calcium binding proteins immunoreactive cell densities				
Toral subdivision	TSc - SG	TSl – SG	TSc – SN	TSl - SN
TSc – SG	–	p = 0.347	p = 0.300	**p = 0.015**
TSl – SG	–	–	p = 0.171	**p = 0.003**
TSc – SN	–	–	–	**p = 0.038**
TSl - SN	–	–	–	–

Bold values indicate significant *p* values.

Having identified a significant interaction and effect of area, we next tested which CBP was responsible for this effect in the overall pattern of CBP-ir cells.

For *calretinin*, linear mixed effect models revealed a significant effect of area (*p* < 0.0001). This indicates that CR-ir cell densities were different between the two toral subdivisions in an area-specific fashion. There was no effect of species (*p* = 0.410) and no interaction between species and area (*p* = 0.107).

For *calbindin*, the linear mixed effect models revealed a significant effect of area (*p* < 0.0001) but no significant interaction (*p* = 0.080) or effect of species (*p* = 0.844). These results indicate that there was a difference in CB-ir cell densities between the two toral subdivisions in both species.

For *parvalbumin*, the linear mixed effect models revealed a significant effect of area (*p* < 0.0001) but no interaction between species and area (*p* = 0.157) and no effect of species (*p* = 0.065). Due to these missing effects in species or interaction we did not perform a *post hoc* bootstrap analysis for any of the individual CBPs tested.

## Discussion

Different signaling modalities can be used for communication. While the use of sound for social signals can be observed in many teleost fishes (Rice et al., 2022), the production of electric discharges for communication is far less common (Crampton, 2019). In this study, we investigated whether an evolutionary shift to a different communication modality is reflected in the central nervous system. Using two closely related catfish species, namely SN and SG, we probed the TS, a central hub of acoustic and electric information processing in the midbrain at macro- (brain region volumes) and microscopical levels toward changes in cell density, volume, connectivity, and immunohistochemical profiling of selected calcium binding proteins. We observed that the central portion of the TS (TSc) was larger than the lateral portion (TSl) in both species. We did not find major changes between the two species in the intensity of DAPI signal (as an indicator for an increase in cell size/cell density). However, we observed changes in the CBP profile in TS regions between SG and SN. Our immunohistochemical results indicate that the electrosensory TSl has different CBP expressions than the TSc in both species. These differences might play a role in enhancing the processing of electrosensory signals, which require fast neuronal firing, especially in electro-communicating species, as previously proposed (Friedman and Kawasaki, 1997). Whether these changes accompanied the development of electrocommunication capability remains to be determined.

### *Synodontis* display a preference toward conspecifics that depends on the sensory cues available

Social recognition is a fundamental step to engage in social communication and can be based in one or many sensory modalities. We evaluated the preference of our investigated species to spend time with conspecifics using a two-choice test. Our *no deprivation* test indicated that both species could be able to differentiate between con- and heterospecifics in the experiments where various sensory cues, including visual, olfactory, auditory and electric cues were present. The *olfactory/electric* cues deprived experiment indicated that, under our experimental conditions, visual/acoustic cues alone do not suffice to elicit a preference for conspecifics in either species. This is especially interesting, considering the known importance of olfactory cues in establishing social preference observed in other catfish species (Slavík et al., 2022).

The auditory playback test revealed that, when presented with auditory stimulation resembling the fundamental frequency of swim bladder tonal sounds (206 Hz), SG exhibited no preference for the conspecific-like tonal sound. This is similar to SN which showed no preference for any of the two sounds presented (206 Hz or broadband noise stimulus). This finding aligns with previous experiments on *Synodontis*, which have demonstrated a negative response to intrusive behavior via swim bladder vocalizations (Abu-Gideiri and Nasr, 1973). SG produces two distinct types of swim bladder sounds: tonal sounds with a fundamental frequency of 206 ± 17 Hz and pulsed sounds with a fundamental frequency of 138 ± 36 Hz (Kéver et al., 2020). It is conceivable that the pulsed sounds may hold different behavioral significance than those shown here for tonal sounds. Future studies should explore whether different *Synodontis* species exhibit a preference for sounds at different frequencies or with different frequency compositions.

Finally, the electric playback experiment revealed a preference for conspecific signals in SN after exposure to a conspecific ED. This preference was absent in SG which only possesses electrosensory abilities, but does not produce EDs for social communication (Kéver et al., 2020). This result is in line with previous experiments that have shown a preference for conspecific EDs in other electro-communicating species, but the absence of this preference if these fish are exposed to other, non-socially relevant, electric signals (Feulner et al., 2009; Nagel et al., 2018). These results might indicate the capacity of SN to use electric signals (in the absence of any other sensory components) to choose conspecifics. The preference toward electrosensory signals in the electro-communicating species could indicate distinct features in electrosensory brain regions that process communication signals such as the TS.

### Conserved structure of the electrosensory and acoustic toral regions

We tested for potential alterations of toral connectivity in the acoustic and electroreceptive neuronal systems of SN and SG. While catfish toral connections and its electrosensory lateral line system have been documented previously (Finger, 1980; Finger and Bullock, 1982; Finger and Tong, 1984; Striedter, 1991; New and He, 1998), adaptations of these sensory systems to different communication modalities remained unstudied. Our results show that *Synodontis*, toral connectivity followed connectivity patterns reported in other fishes, including catfishes (Finger and Tong, 1984; Striedter, 1991) (Figures 3H, 4H for schematic connections found in this study). However, we found stronger projections from the TSl of SN to the nPE (Figure 3 F.1), a relay station of electrosensory processing located between the ELL and the torus (Finger and Tong, 1984; Bastian and Bratton, 1990; von der Emde and Bell, 1996). Stronger input to the TSl from AT was also found in SN in comparison to SG, in which only a few cells projected to the lateral torus (Figure 3 B.2 for SG and Figure 3 E.2 for SN). AT is an important diencephalic region that processes, among others, both acoustic and electrosensory signals (Striedter, 1991) and its role in social communication has been studied in vocal and electric species (Giassi et al., 2007; Harvey-Girard et al., 2010; Petersen et al., 2013). AT is part of the social behavior network as shown by mRNA expression and electrophysiological studies in midshipman and rainbow trout (*Oncorhynchus mykiss*) (Goodson and Bass, 2000; Folgueira et al., 2004; Forlano et al., 2005; Forlano et al., 2010; Petersen et al., 2013). Stronger connections between the electrosensory torus and AT might thus indicate a stronger interface between the electrosensory circuit in SN and brain areas involved social behavior (O’Connell and Hofmann, 2011). Enhanced inputs from this region to the torus might play a role in the processing of electric communication signals in SN. Interestingly, no differences were found in the extent of acoustic input from TSc to AT (Figure 4 B.2 for SG, Figure 4 E.2 for SN).

### No enlargement of the electrosensory TSl in the electrocommunicating SN

We found no differences in size (based on histological sections) of the lateral torus between SG and SN which might indicate that adaptations to an electrocommunication system did not result in an enlargement of this toral division. Interestingly, we found higher levels of DAPI intensity, and thus indirectly of the number of cells or the size of cells present, in the lateral torus of both species in comparison to the central torus. This is most likely a general feature of electrosensation in *Synodontis*. Electrosensation might require more or larger cells to cope with the fast signaling that characterizes this sensory modality (Knudsen, 1976b). Whether there are physiological adaptations to electric signal processing in electro-communicating catfishes thus remains to be investigated.

We found an enlargement of the TSc in both SG and SN compared to their respective TSl, but no differences in the size of the TSl between both species. This indicates that there is no apparent specialization of these regions, that accompanied the change in communication modality. These results leave out the possibility, that neuronal adaptations to electrocommunication could be present at the neurochemical level, that potential adaptations to electrocommunication occurred before electric signals reach the TSl, or that the passive electrosensing networks, present in all *Synodontis* catfishes, were sufficiently adapted to process electrocommunication signals.

### Differential CBPs expression in the TS

Neurochemical profiles underlying the neural acoustic and electrosensory systems involve many different proteins and neuromodulators (Cuadrado et al., 1992; Friedman and Kawasaki, 1997; Castro et al., 2006; Graña et al., 2012). Different CBP profiles have been found in sensory-related regions of the torus in different teleost species (Friedman and Kawasaki, 1997; Castro et al., 2006; Graña et al., 2012). Such changes in CBP expression could alter neuronal firing and excitability, and thus contribute to different sensory processing. While the exact physiological functions of PV, CB, and CR remain not fully elucidated, studies have consistently shown that these proteins, individually or in combination, serve as valuable markers for distinct neuron populations within the vertebrate central nervous system (Arai et al., 1991; Baimbridge et al., 1992; Résibois and Rogers, 1992; Andressen et al., 1993; Friedman and Kawasaki, 1997; Castro et al., 2006; Graña et al., 2012; Kress et al., 2015; Berg et al., 2018).

In this study, we found an increased pattern of CB-ir cells in the auditory torus and enhanced immunoreactivity to CR and PV in the electrosensory torus of both species (Figures 5A, C). The higher levels of CR-ir and PV-ir cells in the lateral torus of both species in comparison to the central torus might indicate a general adaptation to electrosensation in electrosensory fish since a similar result has been previously reported in weakly electric fish (Friedman and Kawasaki, 1997). Based on these results, different CBPs might have different importance in processing of different sensory modalities. CR and PV could be especially important in modulating signal processing in electrosensory processing, while CB might play a more important role in auditory processing. Thus, our individual CBP results do not indicate changes related to the shift in communication modality but instead reveal neurochemical adaptations in two distinct sensory systems in *Synodontis*.

## Conclusion

Our results indicate that both *Synodontis* species prefer conspecifics, and that species preference depend on the presence of communication signal(s). The difference in communication modality between the two species does not correlate with the observed structural and neurochemical changes. Our observed differences instead reflect differences in the organization of the auditory and electric processing torus, common to both species. This does not exclude that SN, may have evolved other adaptations, after its change in communication modality. Such changes could be the observed strengthened connections between TSl and AT, an increased size of other brain regions not investigated here, or changes in the neurochemical profile of electrosensory neurons.

## Data Availability

The raw data supporting the conclusions of this article will be made available by the authors, without undue reservation.

## Footnotes

1
www.tropicwater.eu

## Supplementary material

Supplementary Figure 1 Photographs of calretinin [CR, **(A)**], calbindin [CB, **(B)**] and parvalbumin [PV, **(C)**] expression in neurons and fibers of the different divisions of the torus semicircularis (TS; top row: TSl; middle row: TSc; bottom row: TSv) of *Synodontis grandiops* (SG) and *S. nigriventris* (SN). Lateral nucleus of the torus semicircularis (TSl), central nucleus of the torus semicircularis (TSc), and ventral nucleus of the torus semicircularis (TSv).
