# Supplementary figures and images for: Variation in behavioral preference and calcium binding expression in two Synodontis catfishes with different communication modalities
Source: Front Neuroanat. 2025 Aug 14;19:1589687. doi: 10.3389/fnana.2025.1589687 (PMC12391105; doi:10.3389/fnana.2025.1589687)

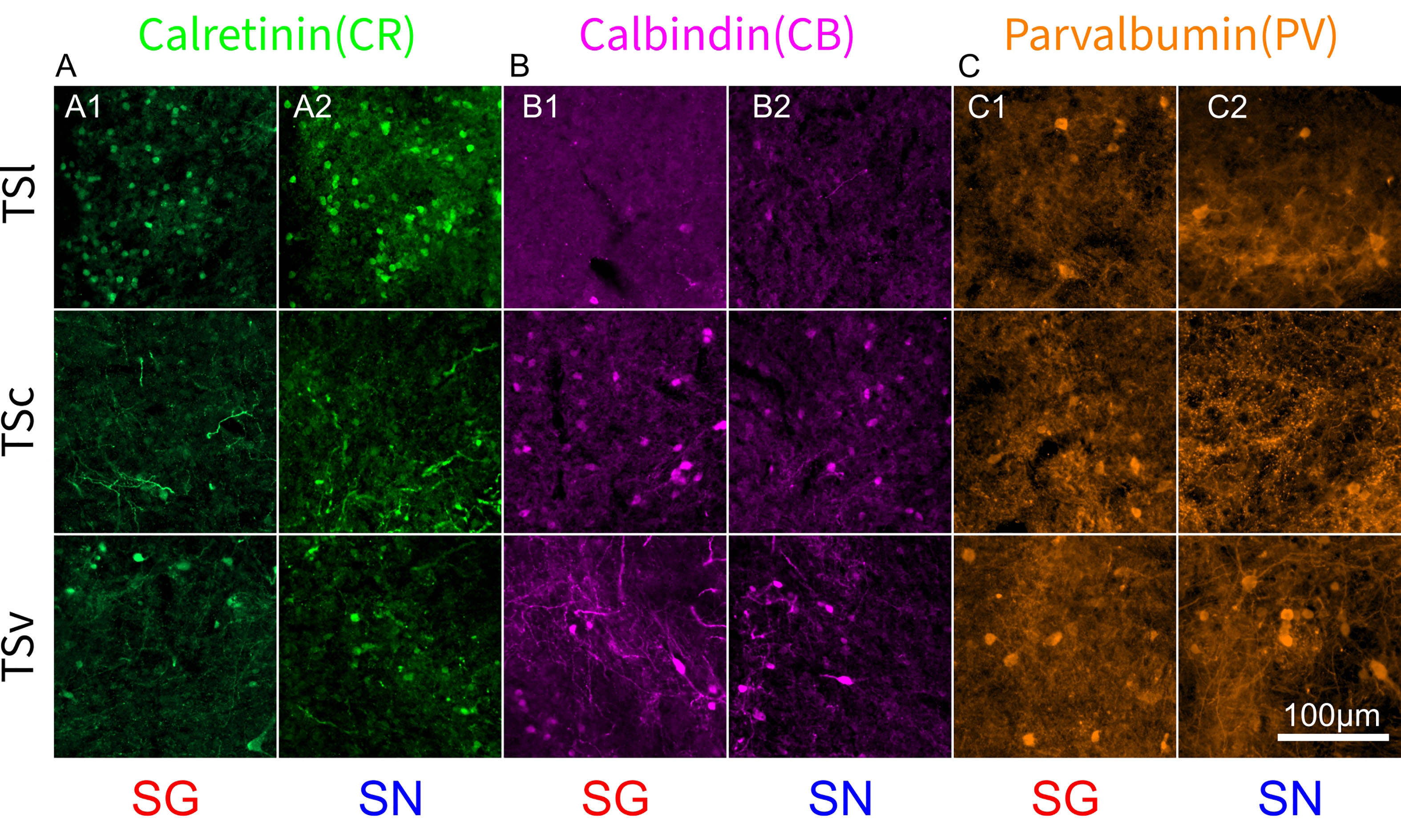

Supplement: Supplementary Figure 1 — Photographs of calretinin [CR, (A)], calbindin [CB, (B)] and parvalbumin [PV, (C)] expression in neurons and fibers of the different divisions of the torus semicircularis (TS; top row: TSl; middle row: TSc; bottom row: TSv) of Synodontis grandiops (SG) and S. nigriventris (SN). Lateral nucleus of the torus semicircularis (TSl), central nucleus of the torus semicircularis (TSc), and ventral nucleus of the torus semicircularis (TSv). [file Image_1.jpeg]
